# Autophagy Flux Contributes to Regulation of Components of *Eclipta prostrata* L. on Cigarette Smoking-Induced Injury of Bronchial Epithelial Cells

**DOI:** 10.3389/fphar.2018.00107

**Published:** 2018-02-20

**Authors:** Shumin Ding, Xuefeng Hou, Gang Wang, Huihui Qiu, Ying Liu, Yuanli Zhou, Mei Du, Xiaobin Tan, Jie Song, Yingjie Wei, Luan Shu, Zhiyong Li, Liang Feng, Xiaobin Jia

**Affiliations:** ^1^School of Pharmaceutical Engineering and Life Science, Changzhou University, Changzhou, China; ^2^Key Laboratory of New Drug Delivery System of Chinese Materia Medica, Jiangsu Provincial Academy of Chinese Medicine, Nanjing, China; ^3^College of Traditional Chinese Medicine, Anhui University of Chinese Medicine, Hefei, China; ^4^China Minority Traditional Medical Center, Minzu University of China, Beijing, China; ^5^College of Traditional Chinese Medicine, China Pharmaceutical University, Nanjing, China; ^6^Affliated Hospital of Long Hua, Shanghai University of Chinese Medicine, Shanghai, China

**Keywords:** *Eclipta prostrata* L., autophagy, cigarette smoking, stress injury, bronchial epithelial cells

## Abstract

Excessive autophagy plays a crucial role in cigarette smoking extract (CSE)-induced inflammation response and oxidative damage of respiratory epithelial cells. The components from *Eclipta prostrata* L. (CCE) have been shown to be beneficial for CSE-induced epithelial cells injury. However, whether its protection on CSE-stress injury is related to its regulation on autophagy remains still unclear. In this study, CCE, containing mainly wedelolactone of 45.88% and demethylwedelolactone of 23.74%, could improve significantly 10%CSE-induced cell viability of normal human bronchial epithelial (NHBE) cells using CCK-8 kit. We revealed that CCE could remarkably increase autophagic factors Beclin-1, Atg5, ATF4 proteins expression levels and the transformation of LC3-I to LC3-II. Additionally, CCE up-regulated significantly p-p16 and p-p21 phosphorylation levels whereas down-regulated p-p53 in NHBE cells. The changes of typical autolysosom and representative autophagosome in the presence of CCE or/and autophagy inhibitor chloroquine (CQ) were also observed by transmission electron microscopy. These data demonstrated that CCE reduced CSE-induced autophagy flux activation in NHBE cells. The blockade of CCE on autophagy flux contributes to its protection against CSE-induced NHBE cells damage, and CCE is promising to be combination therapeutic molecules to excessive autophagic damage in respiratory diseases.

## Introduction

Cigarette smoking has been regarded as a high risk factor in the pathogenesis of lung diseases, and plays a contributor to the molecular events in carcinogenesis of bronchial epithelial cells (Nyunoya et al., 2014). Evidence showed that the stress injury caused by cigarette smoking condensate is thought to be related to the imbalance of homeostasis of normal lung epithelial cells (Yu et al., 2016). Cigarette smoking stimulates normal human bronchial epithelial (NHBE) cells which cause a multistep process involving morphologic and molecular modifications. Then, a complex of alterations occur gradually and lead to malignant transformation with unregulated clonal expansion and cellular proliferation (Gray et al., 2007). The cigarette smoke-induced stress injury is involved in oxidative stress, inflammation response, autophagy activation, and so on. Continuous cigarette smoking increases the probability to absolute mortality risk of various lung diseases for individuals, leading to greater public health problems (Lewer et al., 2017).

Accumulating evidence demonstrates that selective autophagy plays a complex role in human diseases where it can have both protective and injurious effects (Mizumura et al., 2014). It is widely believed that basal or moderate level of autophagy plays a protective role while uncontrolled or overstimulated autophagy may intermediate cell death. Autophagy is reported to be involved in the initiation and pathogenesis of various diseases (Levine and Kroemer, 2008). Such as acute lung injury(ALI), lung cancer, fibrotic lung disease, bronchitis, emphysema, and chronic obstructive pulmonary disease (COPD) (Ryter and Choi, 2015). Li et al. (2016) demonstrated that CSE-induced autophagy in bronchial epithelia, and autophagic gene Beclin-1 and Atg5 were involved in this pathogenesis. Autophagy serves a homeostatic function of lung epithelial cells, contributing to the pathogenesis of smoke-induced carcinogenesis by regulating epithelial cell death. Exposure to cigarette smoking may activate autophagy of stressed-epithelial cells through lysosomal degradation pathways to remove damaged organelles or denatured proteins (Ryter and Choi, 2010). Autophagy induced by inhaled xenobiotics such as cigarette smoke plays an important role in tumorigenesis of respiratory epithelial cells. Many studies showed that heavy smoking may induce some autophagy-related markers such as Beclin 1 in human NSCLC cell lines and NSCLC specimens (Wang et al., 2015; Lv et al., 2015). It is well documented that autophagosomes and p62, a marker of autophagic flux, was observed in smokers’ alveolar macrophages (Monick et al., 2010). The dysfunction of autophagy is considered to be associated with tumorigenesis of lung epithelial cells.

*Eclipta prostrata* L., (Asteraceae), a medicinal herb, has been used in China and Asian countries for both food and medicine purposes for a long time. The traditional function of *Eclipta prostrata* L. focuses on nourishing liver and kidney, cooling blood and hemostasis. Experimental evidence showed that some specific compounds or extracts from *Eclipta prostrata* L. have potential anti-tumor activity (Liu et al., 2012). Recent report showed that the component of *Eclipta prostrata* L. (CCE) is beneficial to treat asthma and other respiratory illnesses (Morel et al., 2017). Interestingly, our previous study showed that the major compound wedelolactone could attenuate CSE-induced oxidant stress and inflammation responses of human bronchial epithelial cells, and screen the active ingredients (Ding et al., 2015). However, whether the protective effect of CCE is related to its regulation on autophagy remains still unclear.

The aim of this study is to explore the regulation of CCE on autophagy of cigarette smoking-induced stress injury of bronchial epithelial cells, and then reveal the possible mechanism. This study provides a beneficial effect and possibility for the heavy smoking-induced injury to bronchial epithelial cells.

## Materials and Methods

### Materials and Reagents

Demethylwedelolactone and wedelolactone (purity ≥ 99%) were purchased from the National Institute for the Food and Drug Control (Beijing, China). Chloroquine was from Sigma–Aldrich (St. Louis, MO, United States). Epigallocatechin gallate (EGCG; ≥98%) was purchased from Nanjing Jingzu Biotechnology Co., Ltd. (Nanjing, China). Beclin-1, LC3-I, LC3-II, Atg5, ATF4, p-p16, p-p21, p-p53 and β-actin antibodies were obtained from Cell Signaling Technologies (Beverly, MA, United States). Secondary anti-horseradish peroxidase goat anti-rabbit IgG, goat anti-mouse IgG were purchased from Beijing Boaosen Biotechnology Co., Ltd. (Beijing, China). Chromatographic grade acetonitrile was purchased from Merck KGaA Co., Ltd. (Darmstadt, Germany). CCK-8 kit was obtained from Nanjing Jiancheng Biotechnology Institute Co., Ltd. (Nanjing, China). DMEM (high glucose) was obtained from Nanjing KeyGen Biotech. Co., Ltd. (Nanjing, China). All other chemicals were of analytical grade and commercial source.

### Cell Culture

Normal human bronchial epithelial (NHBE) cells were obtained from American Type Culture Collection (Rockville, MD, United States) and cultured according to our previous method (Ding et al., 2015). Cells were grown in DMEM (high glucose) containing 10% fetal bovine serum (Gibco, Carlsbad, CA, United States) and 1% penicillin-streptomycin at 37°C in 5% (v/v) CO_2_ atmosphere. After being digested, cells were used for further experiments.

### Preparation of CSE

Cigarette smoking extract (CSE) was prepared according to the method as previously described (Ding et al., 2015). In the present study, Nanjing brand cigarette was used for inducing cells injury. One non-filtered cigarette contains 11 mg tar, 1.1 mg nicotine and 12 mg carbon monoxide. Thirty minutes before the experiment, CSE was prepared by igniting for smoke production. The collection of the produced smoke was performed in a vacuum pump at a rate of 5 min/cigarette. Smoke was dissolved in 10 mL of serum-free DMEM medium and then filtered through 0.22 μm filter membrane.

### Preparation of CCE

The whole herb of Asteraceae was purchased from Meizhou, Guangdong Province, China. The pharmacognostic identification of these samples were identified as *Eclipta prostrata* L. by Prof. Xiaobin Tan in our lab. The plant specimens were deposited at plant sample storage room of Jiangsu Provincial Academy of Chinese Medicine (Store number: EE20160920). The dry herb of 200 g was weighed and then extracted with 4000 mL 70% ethanol for two times (each 2 h). The extract is filtered, combined and removed solvent in a rotary evaporator to obtain *Eclipta prostrata* L. The pigment in extract was removed for four times with an equal volume of petroleum ether. The water fraction was concentrated under pressure and dried at 60°C. The obtained fraction was dissolved in water and then the pH value was adjusted to 4 ∼ 5 with glacial acetic acid. The extraction was eluted by 5 BV 10% ethanol and 5 BV 30% ethanol in D101 macroporous resin. The 30% ethanol eluate was collected and the solvent was recovered under reduced pressure. The resulting solid was recrystallized with methanol and then obtain CCE (Demethylwedelolactone: 23.74%, wedelolactone: 45.88%; the yield was 0.2826%).

### HPLC Analysis for CCE

High performance liquid chromatography (HPLC) was performed on Agilent 1200 HPLC apparatus (Agilent Technologies, Lexington, MA, United States). This apparatus contains a diode array detector (DAD), autosampler, quaternary pump, column heater–cooler and Agilent chemstation software. The analysis conditions was conducted as follows: Agilent ZORBAX 5 TC-C18 (250 mm × 4.6 mm, 5 μm), column temperature: 30°C, detection wavelength: 350 nm, flow rate: 1.0 mL/min; injection volume: 20 μL. Mobile phase consisted of acetonitrile (A) and 0.5% acetic acid-water solution (B). Gradient elution conditions: 0–15 min, 13–18% A; 15–50 min, 18–22% A; 50–65 min, 22–26% A; 65–75 min, 26–46% A; 75–80 min, 46–66% A.

### CCK-8 for Cell Viability

CCK-8 kit (Nanjing Jiancheng Biotechnology Institute Co., Ltd., Nanjing, China) was performed according to the manufacturer’s protocols. NHBE cells (1 × 10^4^ cells/well) were seeded into 96-well plates and treated with CCE and 10%CSE for 24 h at 37°C. Sequentially, CCK-8 of 10 μL was diluted in 100 μL DMEM and then added to each well. After incubation for 4 h, the absorbance of sample was detected using a 96-well plate reader (Molecular Devices, United States) at 450 nm.

### Western Blotting

After being treated with 10% CSE or/and CCE, cells were washed twice with an ice-cold PBS buffer and then were prepared to cell lysates in RIPA lysis buffer. The quantification for protein concentration was determined by BCA Protein Assay Kit. Each aliquot of 20 μg total protein was loaded to electrophoresis on a polyacrylamide gel containing 0.1% SDS (SDS-PAGE). After being transferred to polyvinylidene difluoride (PVDF) membranes (Millipore, Bedford, MA, United States), membranes were blocked with 5% non-fat milk for 1 h at room temperature and incubated with Beclin-1 (1: 500), LC3-I (1: 500), LC3-II (1: 500), Atg 5 (1: 500), ATF4 (1: 500), p-p16 (1: 500), p-p21 (1: 500) and p-p53 (1: 500) primary antibodies overnight at 4°C. β-actin was used as loading control. The membranes were incubated with horseradish peroxidase-conjugated secondary antibody (1: 2000) for 1 h at room temperature. The immunoreactive bands were visualized using an enhanced chemiluminescence (ECL) detection kit and the quantitative analysis of proteins was performed by Image pro plus (IPP) software.

### Transmission Electron Microscopy

Cell suspension was treated with pre-cooled 2.5% glutaric acid and fixed overnight at 4°C for 90 min. After being washed with PBS for three times, cell suspension was fixed with 1% osmium tetroxide at 4°C for 30 min. The cells were dehydrated with 50–100% (10% gradient) of ethanol and pure acetone after PBS washing, and then embedded with Epon812 epoxy resin. The inserts were cut into ultrathin sections (60–70 nm) with microtome and post-stained with uranyl acetate and lead citrate. Finally, the ultrastructural changes of these sections were observed under transmission electron microscope (JEM-1400, JEOL, Japan).

### Autophagic Flux Detection Using mRFP-GFP-LC3 Adenoviral Vector

Autophagic flux was detected using mRFP-GFP-LC3 double fluorescence system (Zhang et al., 2016). Ad-mRFP-GFP-LC3 was purchased from Shanghai Genechem Co., LTD. (Shanghai, China). The detailed operation process of adenoviral infection was performed according to the manufacturer’s protocols. Briefly, NHBE cells on the coverslips at a desired confluency of 50–70% were cultured in DMEM supplemented with 2% FBS with mRFP-GFP-LC3 lentivirus. After 24 h of infection, cells were treated with rapamycin (200 nmol/L) in medium with 10% FBS for 3 h. Autophagy was observed under a confocal laser scanning microscope (Olympus OLS4100, Japan) and confocal images were obtained at 561 and 488 nm.

### Statistical Analysis

All data were presented as means ± standard deviation (SD). The statistical differences were analyzed by SPSS 16.0 software (Chicago, IL, United States) with One-way analysis of variance (ANOVA), using a Bonferroni *post hoc* test. *P* values less than 0.05 were regarded as statistically significant.

## Results

### HPLC Analysis for CCE Composition

**FIGURE 1:**
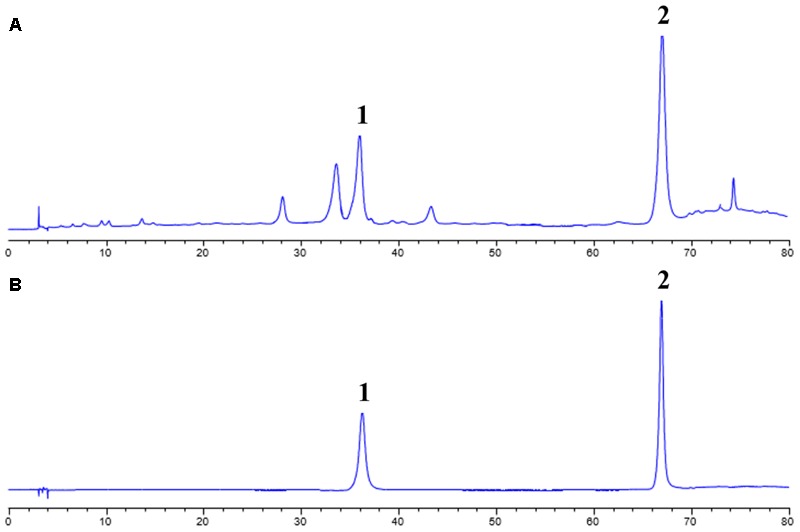
High performance liquid chromatography (HPLC) analysis for CCE **(A)** and mixed reference substance **(B)**. This analysis was performed at 350 nm detection wavelength with mobile phase of acetonitrile and 0.5% acetic acid-water solution.

In order to identify the composition of the isolated CCE, HPLC method was used to analyze the including compounds. As depicted in **Figure 1**, CCE component contains mainly wedelolactone of 45.88% and demethylwedelolactone of 23.74%, when compared with the mixed reference substance. The purity of CCE is 69.62%. The stable component was used for further experiments.

### CCK-8 for Cell Viability

**FIGURE 2:**
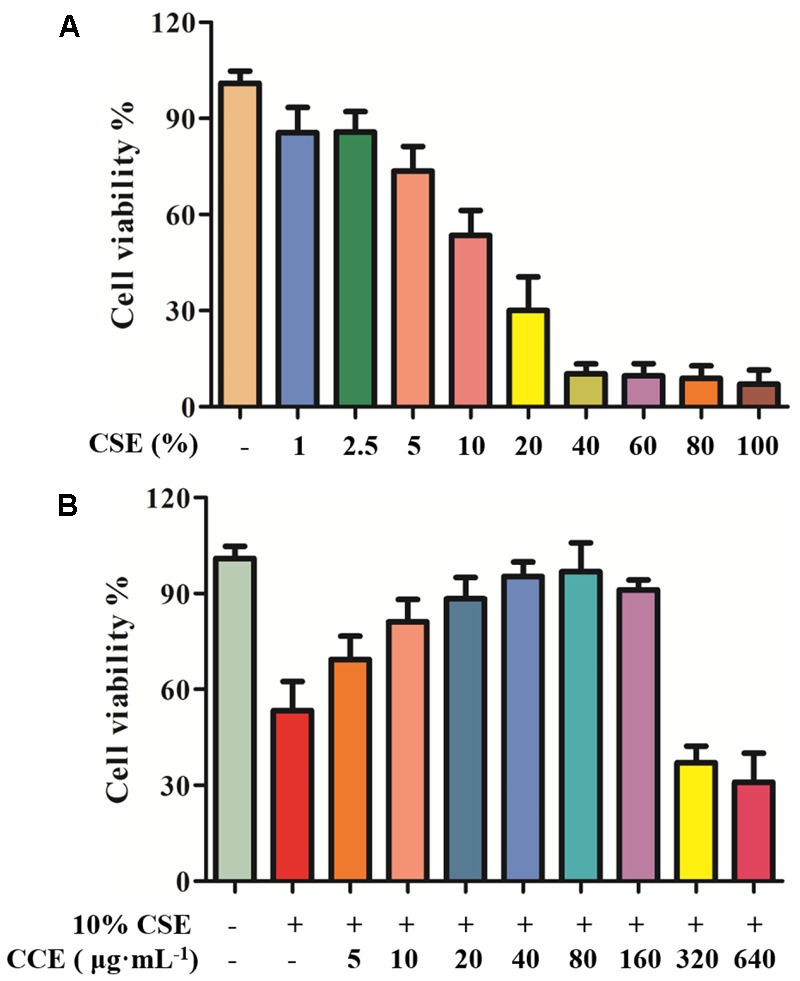
Effects of CCE on cell viability of CSE-induced NHBE cells. CCK-8 assay was conducted to measure the cell viability after being exposed to CSE **(A)** with or without CCE for 24 h **(B)**. The optimal concentrations of CSE and CCE were chosen for further experiments. The absorbance values of samples were detected in microplate. Data are expressed as means ± SD (*n* = 6).

In our previous study, the cell viability of CSE and CCE on NHBE cells was evaluated by CCK-8 assay (Ding et al., 2015). In the present study, CCK-8 method was used to examine the cell viability of CSE and CCE on NHBE cells. As shown in **Figure 2A**, the 24 h exposure of cells to CSE of 1, 2.5, 5, 10, 20, 40, 60, 80, and 100% result in a significant decrease of cell viability. After considering the effect of CSE on cell viability, CSE of 10% was taken as an optimal concentration for further experiments. The bioactivity of CCE at the concentration of 5, 10, 20, 40, 80, 160, 320, 640 μg⋅mL^-1^ was compared in the presence of 10% CSE. **Figure 2B** showed that 80 μg⋅mL^-1^ of CCE has a better effect on CSE-induced NHBE cell injury. Therefore, 80 μg⋅mL^-1^ was chosen as a suitable concentration of CCE for following experiments.

### CCE Reduces Autophagy Level of CSE-Induced NHBE Cells

**FIGURE 3:**
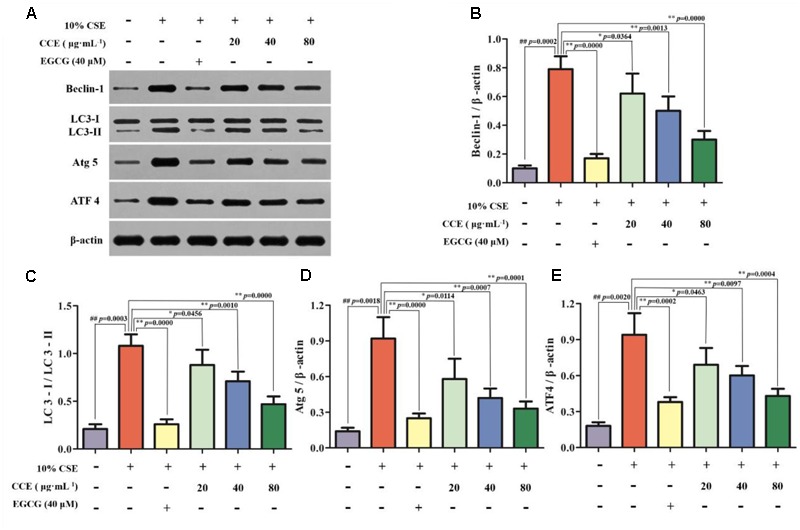
Regulation of CCE on 10%CSE-induced autophagic protein expression levels in NHBE cells. These cells were treated with 10% CSE in the presence or absence of 20, 40, 80 μg⋅mL^-1^ CCE for 24 h. EGCG of 40 μM was selected as positive control. Western blotting analysis **(A)** was performed for the visualization of autophagic proteins including Beclin-1 **(B)**, LC3-II/LC3-I ratio **(C)**, Atg5 **(D)** and ATF4 **(E)**. Data are expressed as means ± SD (*n* = 3). ^##^*p* < 0.01, vs. the blank control; ^∗^*p* < 0.05, ^∗∗^*p* < 0.01, vs. 10%CSE.

Autopahgy plays an important role in the pathogenesis of CSE-induced NHBE cells. Herein, we observed that the stimulation of 10% CSE could up-regulate the autophagic level of NHBE cells through increasing Beclin-1, Atg5, ATF4 and LC3-II/LC3-I ratio. Interestingly, a significant decrease of enhanced autophagic proteins was observed by the treatment of 20, 40, and 80 μg⋅mL^-1^ CCE or positive control 40 μM EGCG (**Figures 3A–E**). The results suggested that CCE could attenuate 10% CSE-induced autophagy of NHBE cells.

### CCE Up-Regulates p-p16, p-p21 and Down-Regulates p-p53 Phosphorylation Levels of NHBE Cells

**FIGURE 4:**
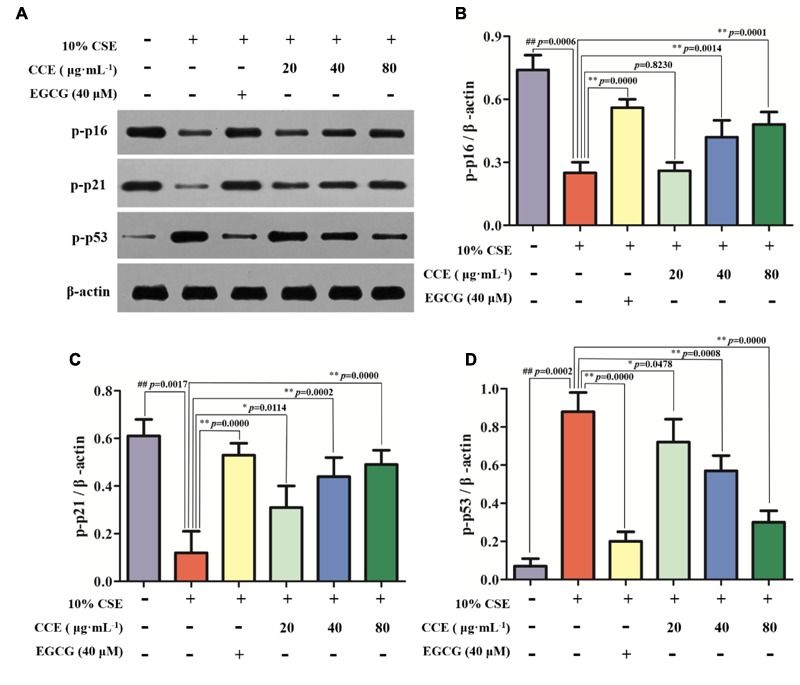
Effects of CCE on the phosphorylation levels of p-p16, p-p21, and p-p53 in 10%CSE-induced NHBE cells. NHBE cells were incubated for 24 h with 10% CSE in the presence or absence of 20, 40, 80 μg⋅mL^-1^ CCE or 40 μM EGCG. **(A)** Protein bands; **(B)** Effect of CCE on the relative expression of p-p16 protein in NHBE cells; **(C)** The relative expression of p-p21 protein; **(D)** p-p35 protein in NHBE cells. Data are expressed as means ± SD (*n* = 3). ^##^*p* < 0.01, vs. the blank control; ^∗^*p* < 0.05, ^∗∗^*p* < 0.01, vs. 10%CSE.

As depicted in **Figure 4**, the exposure of NHBE cells to 10% CSE could decrease the phosphorylation levels of p16 and p21, whereas increase the phosphorylation levels of p53. However, 20, 40, and 80 μg⋅mL^-1^ CCE or 40 μM EGCG could reverse this trend (**Figures 4A–D**). It is worth noting that the regulation of CCE on p16, p21 and p53 phosphorylation levels in a dose-dependent manner. These results indicated that the effect of CCE on CSE-induced autophagic activation might be associated with the phosphorylation of p16, p21, and p53.

### Autophagic Inhibition of CQ Blocked Autophagy Level of CSE-Induced NHBE Cells

**FIGURE 5:**
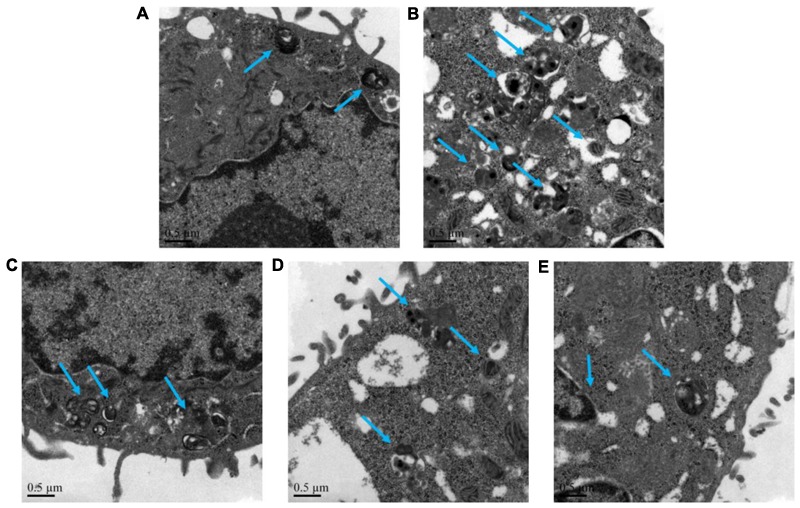
Effects of CCE on autophagy flux of NHBE cells. Transmission electron microscopy was performed to observe the typical autolysosom and representative autophagosome (blue arrows). Autophagy inhibitor chloroquine (CQ) was used to inhibit the excessive autophagy. **(A)** Blank control; **(B)** 10% CSE; **(C)** CCE (80 μg⋅mL^-1^) + 10% CSE; **(D)** CQ (20 μM) + 10% CSE; **(E)** CCE (80 μg⋅mL^-1^) + CQ (20 μM) + 10% CSE. Magnification × 60000.

**FIGURE 6:**
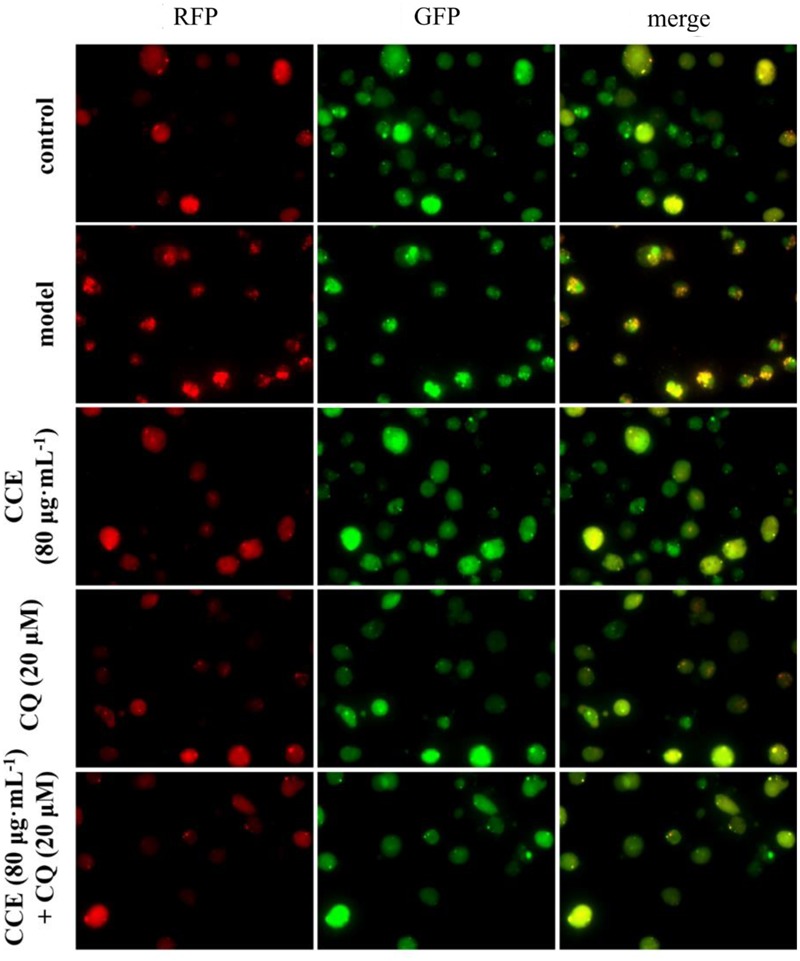
Effects of CCE on autophagy flux of NHBE cells by laser confocal microscopy. mRFP-GFP-LC3 double fluorescence system was used for this determination. Cells were treated with an mRFP-GFP-LC3 lentivirus for 24 h and then treated with rapamycin (200 nmol⋅L^-1^) for 3 h. The red fluorescent represents the formation of autolysosom while green fluorescent represents the formation of autophagosomes.

The autophagy level of NHBE cells was significantly enhanced by the exposure of 10% CSE. As shown in **Figures 5, 6**, typical autolysosom and representative autophagosome can be observed in cytoplasm of 10% CSE-induced NHBE cells under transmission electron microscopy and confocal laser scanning microscopy. In order to explore the regulation of CCE on autophagic flux of NHBE cells, autophagy inhibitor chloroquine (CQ) was adopted to observe the change of autophagy flux. NHBE cells were also pre-incubated with autophagy inhibitor CQ of 20 μM for 1 h followed by 80 μg⋅mL^-1^ CCE or not. Notably, the co-treatment of CCE and CQ could block excessive autophagy flux caused by 10%CSE. These data suggested that the amelioration of CCE on CSE-induced NHBE cells injury may be associated with blocking autophagy flux.

### Effect of Blocking Autophagy Flux on CCE-Mediated Autophagy-Related Proteins

**FIGURE 7:**
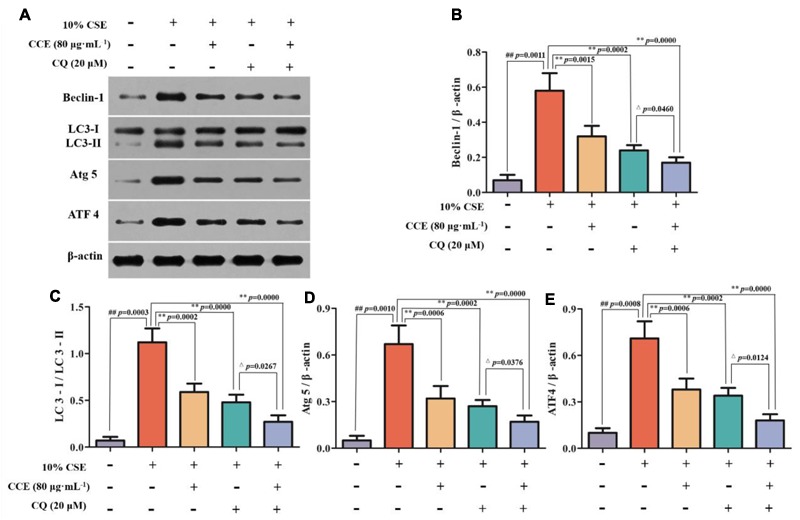
Regulation of CCE on autophagic proteins in the presence of autophagy inhibitor CQ. Cells were treated with 20 mM CQ with or without 80 mg mL^-1^ CCE **(A)**. Autophagic proteins including Beclin-1 **(B)**, LC3-II/LC3-I ratio **(C)**, Atg5 **(D)** and ATF4 **(E)** were detected by Western blotting analysis. Data are expressed as means ± SD (*n* = 3). ^##^*p* < 0.01, vs. the blank control; ^∗∗^*p* < 0.01, vs. 10% CSE; ^△^*p* < 0.05, ^∗∗^*p* < 0.01, vs. 20 μM CQ.

**FIGURE 8:**
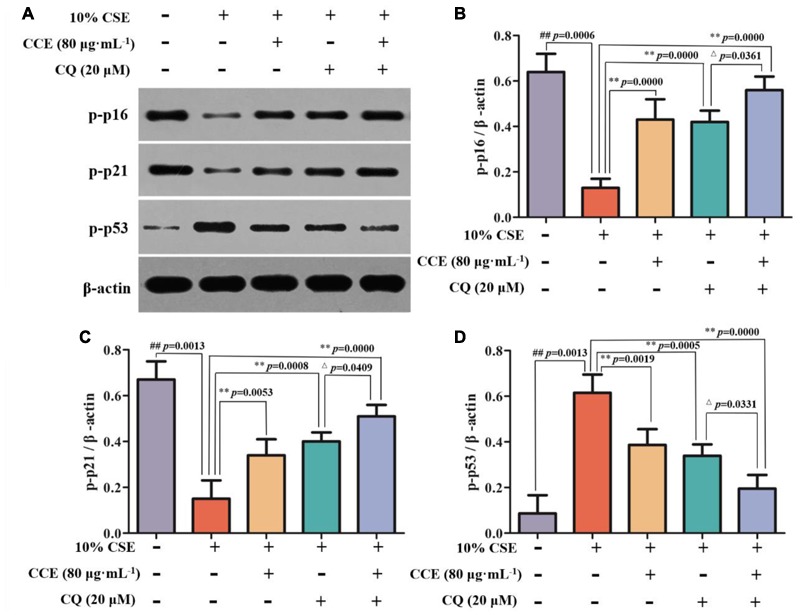
Effects of CCE on the phosphorylation levels of p-p16 **(B)**, p-p21 **(C)** and p-p53 **(D)** in the presence of autophagy inhibitor CQ **(A)**. Data are expressed as means ± SD (*n* = 3). ^##^*p* < 0.01, vs. the blank control; ^∗∗^*p* < 0.01, vs. 10% CSE; ^△^*p* < 0.05, vs. 20 μM CQ.

CSE-induced autophagy contributes to the pathogenesis of respiratory epithelial cell damage. The blockade of autophagy inhibitor CQ was used to observe the changes of autophagy-related proteins. In the present study, CCE or CQ decreased significantly impressed expression of membrane-bound LC3-II, and cytoplasm Beclin-1, Atg5 and ATF4 levels, when compared with 10% CSE (**Figures 7A–E**). Importantly, the co-treatment of CQ and CCE has a significant decrease on these proteins, compared with the alone one. Additionally, the co-treatment of CQ and CCE has also a remarkable regulation on autophagy flux with increasing the phosphorylation levels of p16, p21 and decreasing p53 (**Figures 8A–D**). All results indicated the blockade of CCE on autophagy flux contributes to its protection against CSE-induced NHBE cells damage.

## Discussion

**FIGURE 9:**
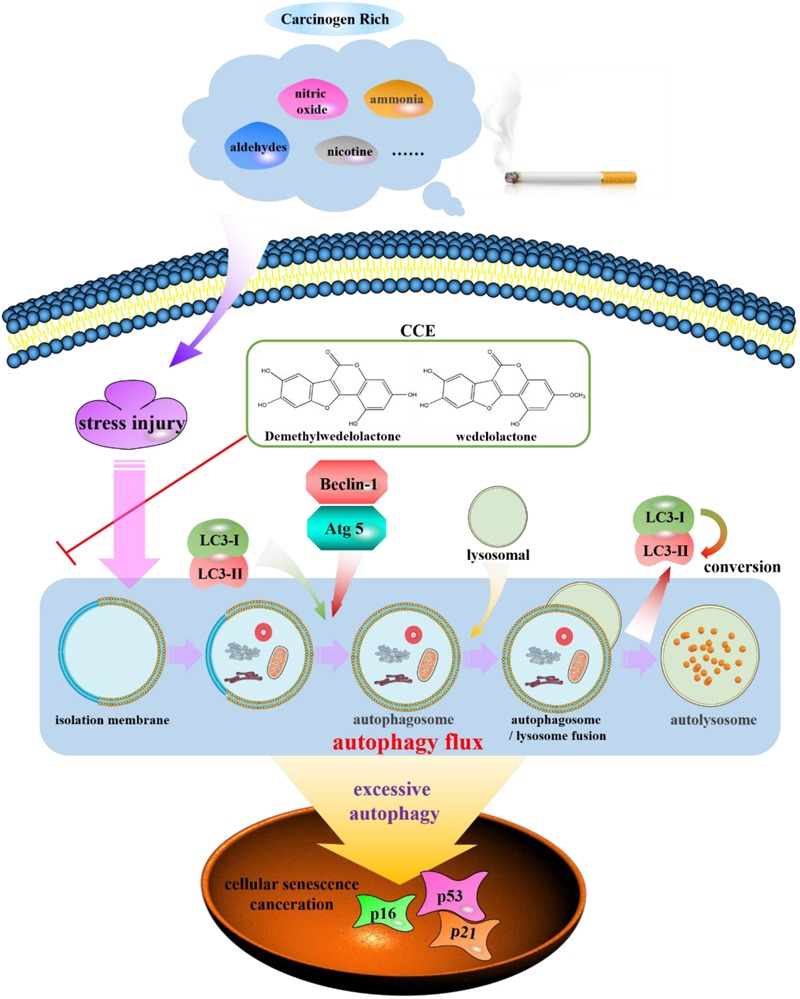
Schematic diagram of autophagy flux contributes to a regulation of CCE on cigarette smoking-induced injury of NHBE cells.

Autophagy, has been found to be a cellular program for organelle and protein turnover and a cell survival mechanism via regulating the sequestration of cytoplasmic contents and orgenelles inside autophagosome (Mizushima and Komatsu, 2011). It has been demonstrated that autophagy plays a critical role in maintenance of cellular homeostasis and the adaption to environmental stress such as oxidative stress, starvation, hypoxia and infection (Gansukh et al., 2016; Zeng et al., 2016; Aseer et al., 2017; Guo et al., 2017). In the present study, the autophagy flux of bronchial epithelial cells exposed to CSE was observed and the regulation of components of *Eclipta prostrata* L. on this function was explored. Our findings indicated that the CCE has a potential down-regulation on CSE-induced excessive autophagic damage of NHBE (**Figure 9**).

*Eclipta prostrata* L., a medicinal herb, has been reported to possess the protection on bronchial hyperresponsiveness and inflammation response (Morel et al., 2017). The findings from phytochemistry showed that coumestan compounds of *Eclipta prostrata* L. are main bioactive components. In this study, we applied D101 macroporous resin to obtain high purity component. The results of HPLC showed this mixed component has a clear and stable composition (wedelolactone: 45.88% and demethylwedelolactone: 23.74%). Our previous report suggested that the main compound wedelolactone from this component possess a protection against CSE-induced oxidant stress and inflammation responses in human bronchial epithelial cells (Ding et al., 2015). Another compound Eclalbasaponin II induces autophagic and apoptotic cell death in human ovarian cancer cells (Cho et al., 2016). The obtain of this component provided a stable and enough compounds for further experiments.

Several studies have focused on the functional link between autophagy and inflammation-associated pulmonary pathogenesis, which suggests a critical role of autophagy in inflammation response and oxidative stress injury, contributing to the pathogenesis of pulmonary diseases like asthma and acute lung injury (Gu et al., 2017; Zeng et al., 2017). Cigarette smoke has been regarded as one of the prime risk factors for chronic lung disease (Li et al., 2016). Under CSE stimulation, the homeostatic turnover of cytoplasmic components of tracheal epithelial cells were required to meet metabolic demands (Li et al., 2017; Sharif et al., 2017). The stimulation of CSE triggers cellular autophagy, causing inflammation response and oxidative stress which are responsible for cell viability. Interestingly, our data suggested that CSE-induced excessive autophagy could decrease the cell viability of NHBE cells. Our results are in agreement with those of the previous work, we reported that 10% CSE was optimal concentration for the cell viability and autophagy of NHBE cells. Of note, the co-treatment of component of *Eclipta prostrata* L.(CCE) and 10%CSE could reverse cell damage and reduce autophagy flux. This might be associated with the attenuation of CCE on CSE-induced autophagic damage of NHBE cells.

The role of autophagy in the mechanisms of respiratory disease has been controversial with studies that have suggested both protective and injurious aspects (Kim et al., 2016). Whether autophagy is protective or detrimental in respiratory disease seems to depend on the extent of its activation, specific stimuli/circumstances, and specific cell type (Ershova et al., 2016). Additionally, the autophagic damage of NHBE cells was related to the stimulation time and concentration of CSE. The optimal incubation condition was screened in our previous and present studies for the excessive autophagic damage(Ding et al., 2015).

To further explore the regulation of CCE on autophagic flux of CSE-induced NHBE cells, we adopted chloroquine, a classical autophagy inhibitor, to observe the change of autophagy flux. In our study, we observed autophagy vacuoles in the cytoplasm of NHBE cells. Abundant autophagosomes, including C-shaped double-membrane structure and round double membrane vacuoles, are visible in NHBE cells under transmission electron microscopy observation, although they were seldom detected in normal cells (Yang et al., 2012). Our data implied that the co-treatment of CCE and CQ could block excessive autophagy flux caused by 10% CSE. This suggested that the reduction of CCE on CSE-induced NHBE cell damage was associated with blocking excessive autophagy.

Autophagy factors play important roles in the cell damage of NHBE cells. The representative and typical autophagy factors Beclin-1, LC3-II/LC3-I ratio, Atg 5 and ATF4 have been shown to act as contributors to the autophagy flux. The evidence showed that the exposure of NHBE cells to CSE could cause the changes of morphological and biochemical markers of autophagy via inducing autophagosome formation (Kim et al., 2008). Herein, we observed the stimulation of CSE increased the processing of microtubule-associated protein-1 light chain-3 (LC3-I) to LC3-II in NHBE cells, and also increased the expression levels of Beclin-1, Atg 5 and ATF4. Contrarily, this up-regulation was reversed by CCE treatment in a dose-dependent manner. Namely, CCE diminished CSE-induced Beclin-1, Atg 5, ATF4 expression levels and partially inhibited the processing of LC3-I to LC3-II. These findings demonstrated an interesting evidence for the regulation of CCE on autophagic and apoptogenic signaling in CSE-induced cell viability.

Recent researches showed that the crosstalk between these autophagy factors might mainly account for excessive activation of NHBE cells because the increasing autophagy triggers inflammatory and oxidative stress microenvironment and other dyshomeostasis. This excessive autophagy is accompanied by activation of both the p16, p21, and p53 pathways. All of these pathways likely contribute to the cell cycle that is typical of cellular senescence (Nyunoya et al., 2014). It has been suggested to function as a natural brake for tumor development and play a critical role in tumor suppression and aging. Recent studies showed that autophagy was able to induce cells entering senescence and eventually lead to cell death (Zhang et al., 2017). Our results showed that CCE could alleviate cell senescence by reducing the autophagy of NHBE cells. Although this result was confirmed, further studies should shed light on the important role of these factors in the protective role of CCE against CSE-induced NHBE cells damage.

In summary, CCE can protect against CSE-induced stress injury of NHBE cells and is likely to take place through reducing excessive autophagy flux. The elucidation of this important interaction between stress injury of NHBE cells and autophagy will advance our understanding of the pathogenic effect of CSE-associated chronic airway diseases and reveal novel drug targets for the development of effective treatments.

## Author Contributions

SD, ZL, XT, LF, and XJ conception and design, acquisition of the data. XH and LF wrote the manuscript. GW, HQ, YL, YZ, ZL, XT, and MD performed the experiments. JS, YW, and LS analyzed the data.

## Conflict of Interest Statement

The authors declare that the research was conducted in the absence of any commercial or financial relationships that could be construed as a potential conflict of interest.
